# Spontaneous Thrombosis and Subsequent Recanalization of a Developmental Venous Anomaly

**DOI:** 10.7759/cureus.334

**Published:** 2015-09-28

**Authors:** Vishal J Patel, Rishi R Lall, Sohum Desai, Aaron Mohanty

**Affiliations:** 1 Division of Neurosurgery, University of Texas Medical Branch at Galveston

**Keywords:** developmental venous anomaly, Venous thromboembolism

## Abstract

Developmental venous anomalies (DVA) are among the most common congenital malformations of the cerebral angioarchitecture. Spontaneous thrombosis of this entity is rare, and our review of the literature found only 31 reported cases of symptomatic spontaneous thrombosis of developmental venous anomalies. Here, we report a unique case describing the spontaneous thrombosis of a DVA leading to venous infarction and subsequent recanalization. The patient was a previously healthy 21-year-old male who presented with an acute onset of partial seizures. Following negative hypercoagulability studies and along with CT (computed tomography) and MR (magnetic resonance) imaging, the patient was treated with anticoagulant therapy and demonstrated complete functional recovery. Knowledge from our literature review of similar cases combined with the experience gained from this patient’s treatment leads us to suggest that spontaneous DVA thrombosis and venous infarction generally has a good outcome despite initially devastating neurologic deficits. Additionally, the rarity of spontaneous DVA thromboses lends itself to the need to identify possible predisposing risk factors, chief amongst these being hypercoagulopathies.

## Introduction

Developmental venous anomalies (DVA), also known as venous angiomas, are malformations of the cerebral venous drainage system. They have a reported incidence of up to 2.5% in post-mortem autopsy, and they account for nearly 55% of all cerebral vascular malformations discovered by radiographic study [1-3]. DVAs are frequently associated other neurovascular malformations, such as cavernous angiomas [1].

Spontaneous thrombosis of this entity is rare but has been reported sporadically in the literature (Table 1). Several reported instances occurred in patients with conditions predisposing to hypercoagulabilities, such as Factor V Leiden mutation, smoking, and oral contraceptive use [4-5]. Although the majority of these lesions are benign, they can incur deficits related to increased arteriovenous shunting or venous congestion. In rare cases, DVAs may thrombose, leading to venous obstruction [6]. Here, we present a unique case of a symptomatic spontaneous DVA thrombosis with subsequent recanalization.

## Case presentation

**Figure 1:**
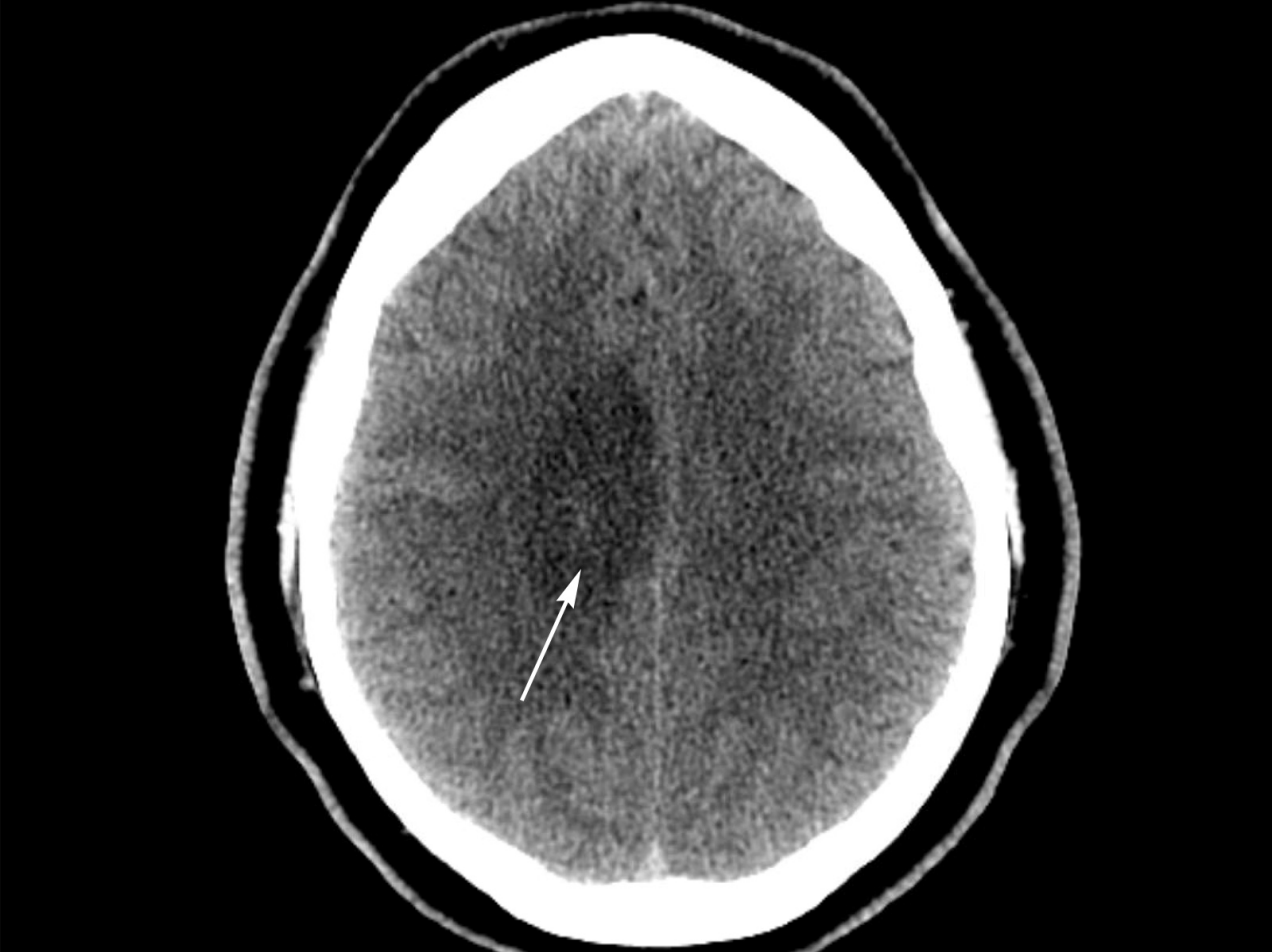
Non-contrast CT head demonstrating vasogenic edema surrounding underlying DVA

A previously healthy 21-year-old male presented with new onset partial seizures consisting of tonic-clonic activity affecting the left lower extremity in addition to numbness in the left upper extremity. His physical examination on presentation was significant for weakness in the left lower extremity, worse distally (1/5) than proximally (3/5). Initial coagulability studies, including PT, INR, and aPTT, were within normal limits (13.9 seconds, 1.0, and 28 seconds, respectively). A CT scan of the head without contrast, shown in Figure 1, revealed a 4.7 cm X 2.4 cm X 1.9 cm hypodensity along the medial aspect of the pre- and post-central gyri without significant mass effect. Informed patient consent was obtained for this patient's treatment.

**Figure 2:**
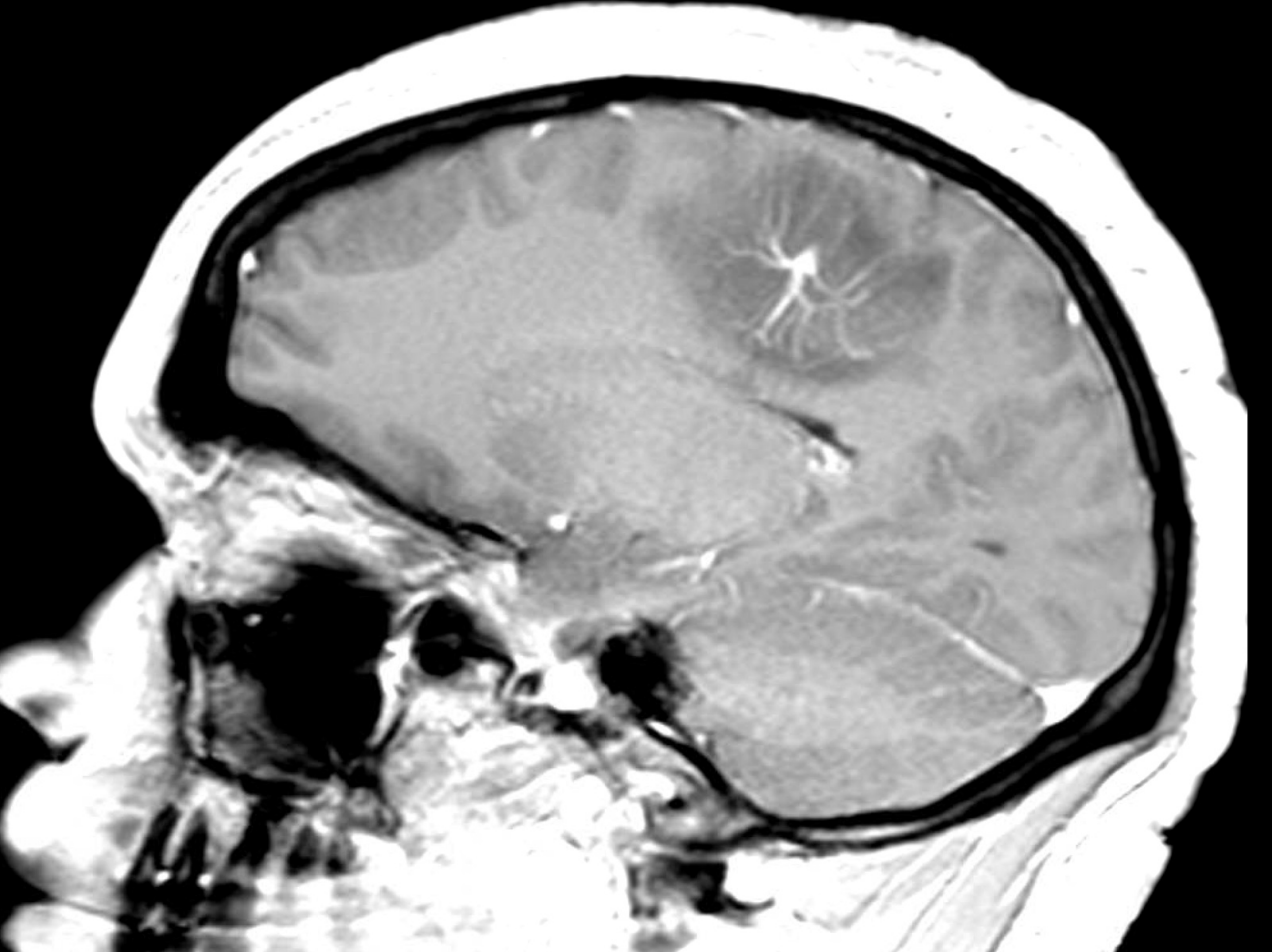
Contrasted MR demonstrating DVA and surrounding vasogenic edema

Given the suspicion for an underlying mass lesion, an MRI with contrast was obtained and demonstrated a prominent developmental venous anomaly draining into the superior sagittal sinus with significant peri-lesional vasogenic edema (Figure 2). No associated cavernous malformation was present.

**Figure 3:**
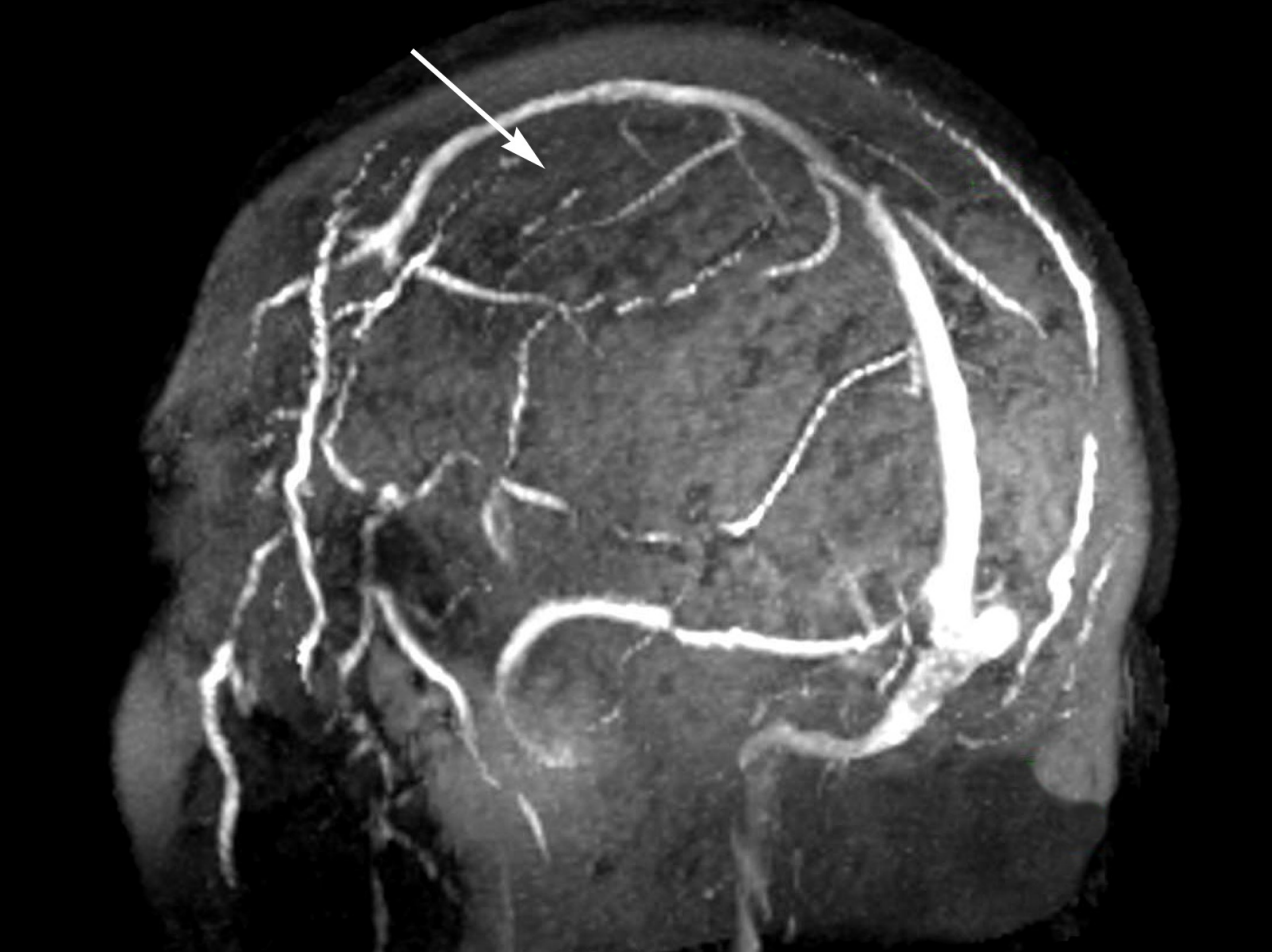
MR venogram demonstrating lack of flow through previously described DVA

MR venography demonstrated non-filling of the venous angioma, suggesting that the draining vein had thrombosed (Figure 3).

**Figure 4:**
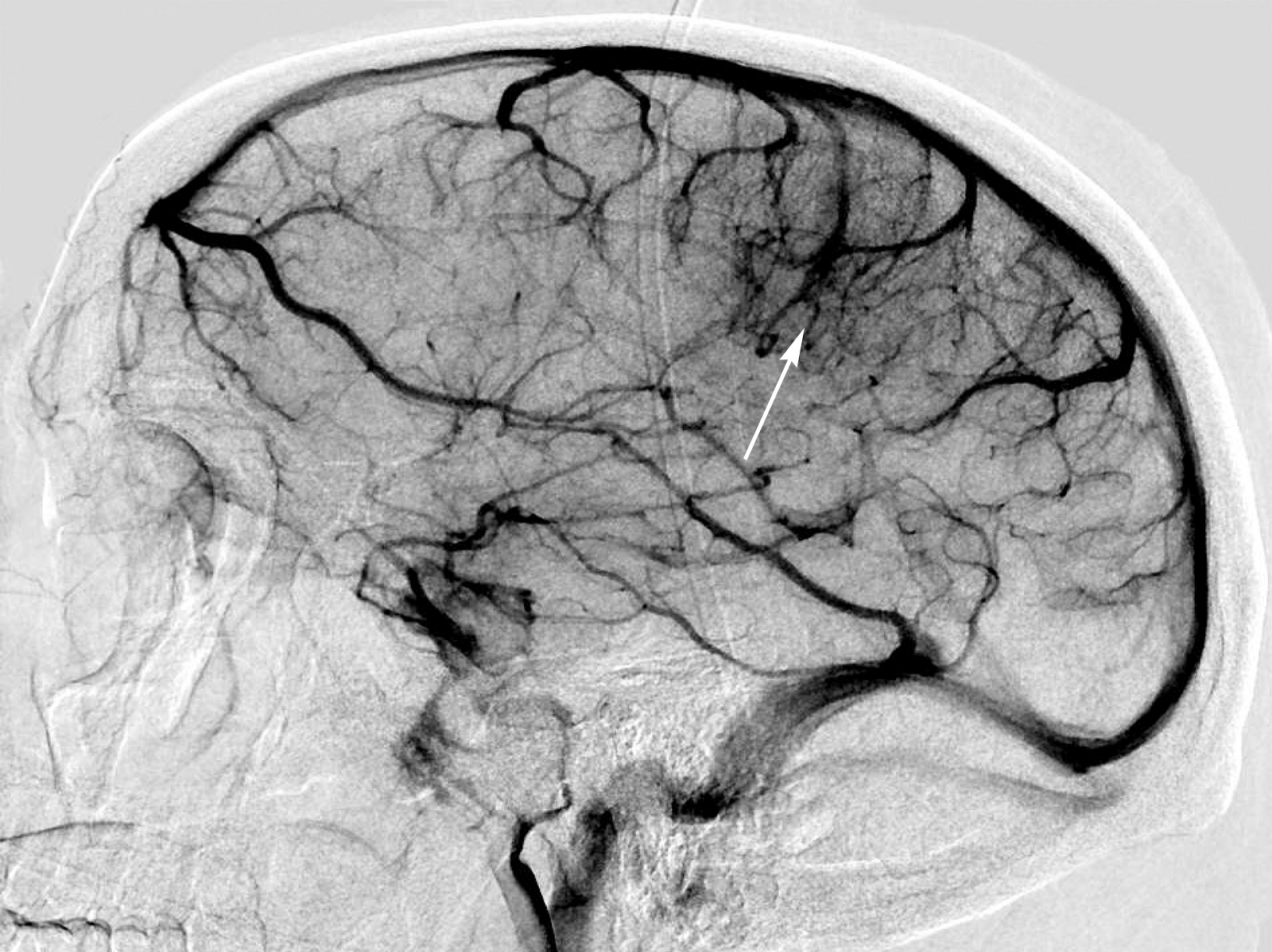
Venous phase right internal carotid DSA demonstrating recanalization of previously mentioned DVA after IV heparin therapy

Following initial coagulability studies and after the collection of antithrombin III deficiency and Factor V Leiden mutation studies, the patient was started on intravenous heparin therapy. A four-vessel cerebral angiogram was then performed to evaluate for any other concurrent vascular lesions, such as a dural AV fistula (Figure 4). The arteriogram revealed the draining vein had recanalized.

By hospital day five, the patient had improved to 4/5 in the proximal left lower extremity but remained 1/5 distally. The patient was then discharged on 81 mg aspirin therapy.

**Figure 5:**
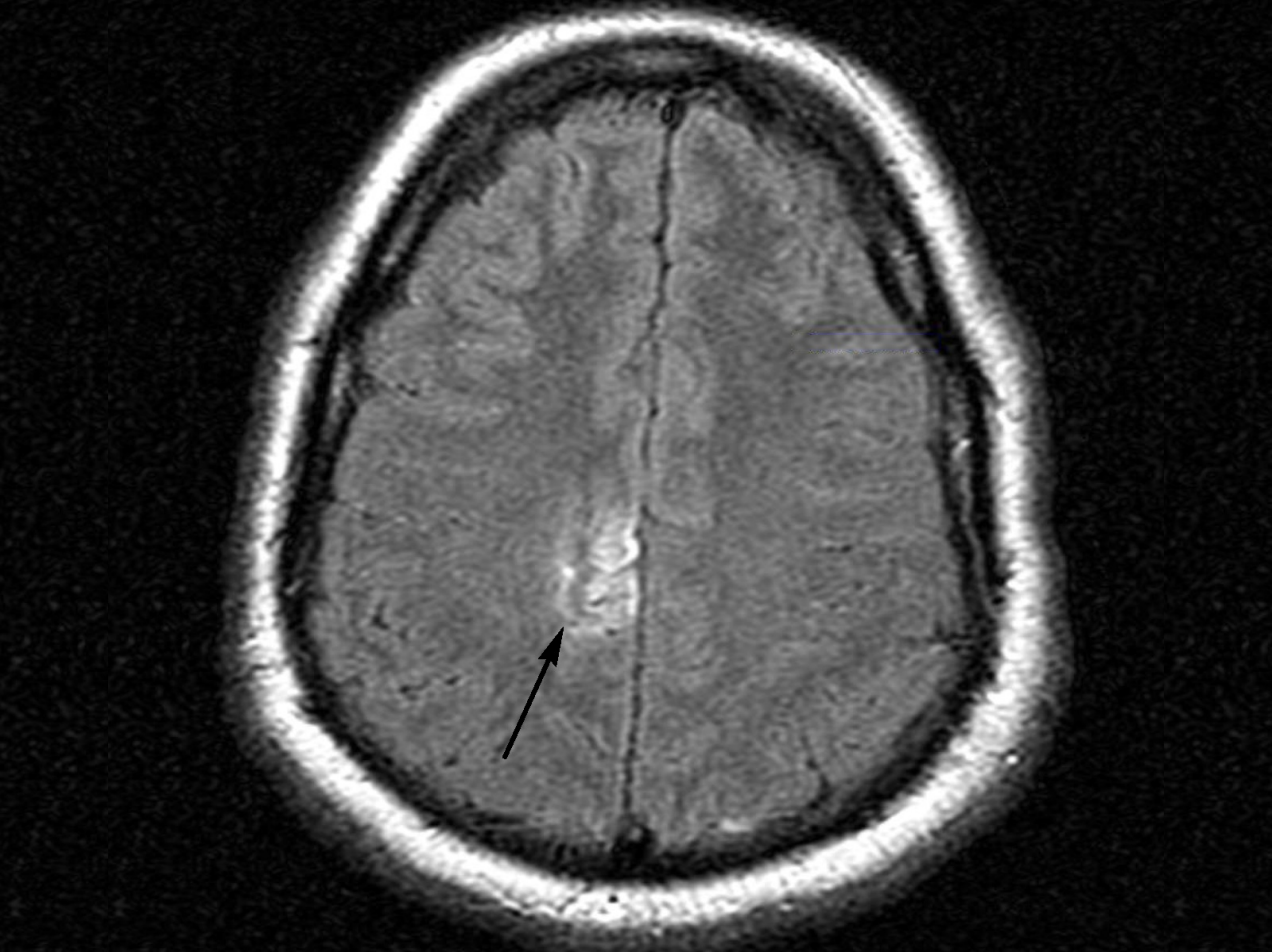
MR FLAIR demonstrating cortical laminar necrosis

Subsequent examination five weeks after presentation demonstrated the patient had complete recovery of sensation in his left upper extremity as well as strength in his left lower extremity. Activated protein C resistance assay for Factor V Leiden mutation (ratio of 2.3) and antithrombin III levels (97%) were eventually found to be within normal limits. His aspirin therapy was discontinued in light of negative anticoagulation workup. A follow-up MRI scan three months post-discharge revealed laminar necrosis; however, his physical exam was negative for any sensory or strength deficits (Figure 5).

The patient remains neurologically intact with no sensory deficits or weakness now one year from his initial presentation.

## Discussion

Spontaneous thrombosis of a developmental venous anomaly is uncommon and has been limited to 31 cases described in the literature (Table 1).

**Table 1:** Reported Cases of Symptomatic Thrombosis of Developmental Venous Anomalies

Author (Year)	Patient	DVA Location	Risk Factors	Management	Outcome
Agarwal, et al. (2014) [7]	61 yo male	Left cerebellar	None reported	Anticoagulation therapy (type not specified)	Improvement with residual mild left-sided ataxia
Yi, et al. (2013) [8]	31 yo male	Left frontal	None reported	Conservative	Improved neurological function, but developed generalized seizures
Griffiths, et al. (2013) [9]	52 yo male	Right pons	None reported	Warfarin	Complete recovery
Su, et al. (2013) [10]	37 yo female	Left frontal	None reported	No treatment initiated	Complete recovery
Pilato, et al. (2013) [11]	62 yo male	Right frontoparietal	None reported	Low-molecular-weight heparin	Complete recovery
Kiroglu, et al. (2011) [12]	36 yo female	Left cerebellar	None reported	Occipital decompression followed by conservative therapy	Good recovery with residual mild left-sided ataxia
Sepelyak, et al. (2010) [5]	17 yo female	Left frontal	heterozygous for Factor V Leiden R506Q mutation and oral contraceptive use	Oral contraceptives discontinued	Good recovery
Toulgoat, et al. (2010) [4]	44 yo female	Left temporal	heterozygous for Factor V Leiden R506Q mutation	Antiepileptic and anticoagulation therapy	Complete recovery
Abarca-Olivas, et al. (2009) [13]	28 yo female	Right frontal	Oral contraceptive use	Craniotomy and partial hematoma evacuation and systemic anticoagulation	Good recovery with residual mild hemiparesis
	38 yo male	Right frontal	None reported	Conservative	Complete recovery
Pereira, et al.(2008) [6]	1 yo female	Left temporal	None reported	Conservative	Unspecified
	20/24 yo male	Bilateral cerebellar	Stenosis of venous collector/blue rubber bleb nevus syndrome	1998 - III ventriculostomy 2002 - Conservative	Unspecified
	1 mo male	Left temporal	Stenosis of venous collector	Conservative	Normal development
	32 yo female	Cerebellum	Thrombosis of venous collector	Conservative	Complete recovery
	8 mo female	Left temporal	Thrombosis of venous collector	Conservative	Good recovery / normal development
	11 mo female	Right cerebellar	Stenosis of venous collector	Conservative	Unspecified
	5 yo female	Right temporal	Thrombosis of venous collector	Conservative	Good recovery
	29 yo male	Left frontal	Stenosis and thrombosis of venous collector	Anticoagulation therapy (type not specified)	Good recovery
	58 yo female	Left cerebellar	Stenosis of draining vein	Anticoagulation therapy (type not specified)	Good recovery
	41 yo male	Right temporal	Left frontal AVM	AVM embolization	Good recovery
	9 yo male	Left temporal	Pseudoaneurysm	Arterial embolization	Good recovery
	14 yo female	Bilateral cerebellar	Microshunts	Arterial embolization	Good recovery
	24 yo male	Right frontal	Microshunts	Arterial embolization	Good recovery
	8 yo male	Right cerebellar	Microshunts	Arterial embolization	Good recovery
	2 day female	Left frontal	Normal	Conservative	Good recovery / normal development
	32 yo female	Left basal ganglia	Normal	Conservative	Good recovery
	42 yo female	Left cerebellar	Normal	Conservative	Unspecified
Konan, et al. (1999) [14]	31 yo male	Bilateral cerebellar	None reported	Conservative	Residual right facial palsy
Merten, et al. (1998) [15]	50 yo female	Left frontal	None reported	Intravenous heparin	Complete recovery
Field and Russell (1995) [16]	34 yo female	Right parietotemporal	None reported	Conservative	Unspecified

The average age of patients was 27.9 years (range: 2 days to 62 years), and there appeared to be no gender predilection. Prognosis generally appears to be good with 73% of reported cases having a good or complete recovery and 83% having improvement of any kind. In our review of the literature, therapy ranged widely from serial observation to anticoagulation.

Notably, though the patient eventually achieved full functional recovery, we observed that clinical improvement lagged behind radiographic resolution. Experience gained from this patient's treatment and from previously reported cases of DVA thrombosis suggests that physicians and family should not despair if clinical improvement is delayed or if presentation is late.

Although developmental venous anomalies are rarely symptomatic, they are common anatomical variants. Most patients with DVAs are told this finding is purely incidental and warrants no major concern. However, we propose a few caveats to this practice. First, spontaneous DVA thrombosis and venous infarction, although rare, should be included on the differential diagnosis for patients with a DVA presenting with new neurologic deficits. Correctly differentiating lesions caused by thrombosed DVAs from other pathologies, such as neoplasms, allows for the quicker initiation of the appropriate therapy. Second, patients with incidentally discovered DVAs should be offered screening for coagulopathies to help assess the risk of thrombosis, especially if the DVA is draining in eloquent territories. Although coagulability workup was unrevealing in this particular patient, the literature contains several instances of a DVA thrombosing in patients with predisposing hypercoagulable states [4-5]. Thrombosis in a young patient with no recent history of trauma, major surgery, or extended immobilization underlies the importance of a hypercoagulability workup to discover any additional risk factors.

## Conclusions

Patients harboring developmental venous anomalies (DVA) should be aware that, although rarely symptomatic, these lesions can thrombose spontaneously and present with neurologic deficit.
